# Fetal Deaths in SARS-CoV-2-Infected Pregnant Women: A Portuguese Case Series

**DOI:** 10.1155/2022/8423733

**Published:** 2022-08-03

**Authors:** Ana Rita Mira, João Pedro Pereira, Catrine Dahlstedt-Ferreira, Margarida Enes, Hélder Oliveira Coelho, Ana Beatriz Godinho

**Affiliations:** ^1^Obstetrics and Gynaecology, Hospital Garcia de Orta, Almada, Portugal; ^2^Pathology, Hospital Garcia de Orta, Almada, Portugal

## Abstract

**Introduction:**

Stillbirth has been documented as an outcome of SARS-CoV-2 infection in pregnancy. Placental hypoperfusion and inflammation secondary to maternal immune response seem to play a role in the cascade of events that contribute to fetal death. The aim of our study is to report a perinatal outcome of SARS-CoV-2 infection in pregnancy adding information to the pool of data on COVID-19 pregnancy outcomes. *Case Presentation*. This is the first stillbirth case series occurring in pregnant women infected with SARS-CoV-2 in a Portuguese cohort. Between April 2020 and March 2021, we had 2680 births in our centre, of which 130 (4.95%) involved mothers infected with SARS-CoV-2. Of total births, there were 14 stillbirths (0.52%), accounting for the highest stillbirth rate we have had in the last 5 years. Among these 14 stillbirths, 5 (35.71%) occurred in SARS-CoV-2-infected mothers. We report the clinical features and placental histopathologic findings of 4 stillbirth cases that occurred in our hospital. *Discussion*. The stillbirth rate among SARS-CoV-2-infected pregnant women (5/130; 3.84%) was significantly increased compared to noninfected patients (9/2550; 0.35%). Most women (3/4) were asymptomatic for COVID-19, a surprising outcome, given the current literature. All cases had histologic exams showing placental signs of vascular malperfusion, although we acknowledge that 3/5 had obstetric conditions related to placental vascular impairment such as preeclampsia and HELLP syndrome.

**Conclusion:**

Stillbirth can be a perinatal consequence of SARS-CoV-2 infection in pregnancy, even in asymptomatic patients. We urge more studies to explore the association between SARS-CoV-2 infection and the risk of stillbirth.

## 1. Introduction

Coronavirus disease 2019 (COVID-19) is primarily a respiratory infection caused by a novel severe acute respiratory syndrome coronavirus-2 (SARS-CoV-2) [1]. Pregnant women are not thought to be more susceptible to the infection than the general population [2]. However, changes to their immune system mean they are more vulnerable to developing severe disease and being admitted for intensive care support [3, 4]. There have been a few maternal risk factors for severe disease and poorer maternal outcomes. These were maternal age above 30 years, BMI above 30 kg/m^2^, mixed ethnicity, and preexisting comorbidities [5–7]. Studies from registries of pregnant women have reported a greater risk of pregnancy and perinatal complications such as preterm birth and neonatal unit admission [5, 8–12]. There is mixed and “context-dependent” evidence on stillbirth as a direct and indirect outcome of the COVID-19 pandemic [13]. While some cohorts did not demonstrate a significant increase in stillbirth rate [7, 8, 14, 15], growing evidence suggests that stillbirth might be a consequence of SARS-CoV-2 infection in pregnancy, especially during the period of Delta variant predominance [5, 9, 16–18]. As an indirect mechanism to explain the increased stillbirth rate, lockdown measures and fear of infection accounted for less access to healthcare, resulting in decreased quality of antenatal surveillance [14, 18]. On the side of direct mechanisms, it is known that SARS-CoV-2 infection triggers a host immune response in the form of a hyperinflammatory “cytokine storm” that causes endothelial dysfunction and coagulation abnormalities [19, 20]. Together, these events result in suboptimal perfusion of vital organs, such as the placenta, ultimately compromising fetal well-being [21]. Indeed, a significant proportion of women with SARS-CoV-2 infection in pregnancy showed placental histopathologic lesions suggesting hypoperfusion and inflammation [22, 23].

This is the first case series of stillbirths occurring in pregnant women infected with SARS-CoV-2 in a Portuguese cohort. We report 4 cases of stillbirth, 3 of which occurred in COVID-19 asymptomatic patients, a surprising outcome, given the current literature [22, 25]. Another interesting aspect of our case series is that all cases had placental signs of vascular malperfusion, a finding that has been associated with SARS-CoV-2 infection. During the COVID-19 pandemic, all women admitted for labour in our institution performed a Polymerase Chain Reaction (PCR) SARS-CoV-2 screening test. Our case report is aimed at contributing to the pool of data on COVID-19 pregnancy outcomes and improving evidence for better obstetric counselling and maternal and perinatal care.

### 1.1. Case Presentation

### 1.2. Overview

Between April 2020 and March 2021, we had 2624 deliveries in our hospital (a tertiary centre), accounting for 2680 births. Of all deliveries, 130 (4.95%) involved mothers infected with SARS-CoV-2 at birth. In this group of positive mothers, 109 (83.84%) were asymptomatic, 15 (11.53%) had mild symptoms of COVID-19, 5 (3.84%) had moderate symptoms, and 3 (2.30%) had severe disease requiring ICU admission. We had 14 stillbirths overall, of which 5 (35.71%) occurred in SARS-CoV-2-infected mothers.  Table 1 summarises the main characteristics of stillbirths that occurred in our centre from April 2020 until March 2021. Categorical variables were expressed in absolute and relative frequencies, and continuous variables were expressed as mean and standard deviation. We performed a proportion difference analysis with IBM SPSS version 26 (Chicago, IL, USA) and set statistical significance at *p* < 0.05. Stillbirth incidence among SARS-CoV-2-positive patients was 3.84% (5/130), whereas stillbirth incidence, in noninfected patients, was 0.35% (9/2550) (${\underset{¯}{\chi }}^{2}\;\left(1\right)=29,046$; *p* < 0.05). Overall stillbirth rate was 0.52% (14/2680) which increased in comparison to the analogous period of the 4 previous years, as seen in Table 2. However, this increase was not statistically significant under a *Z*-test comparing the differences between the stillbirth rate of the period between April 2020 and March 2021 and each of the previous analogous periods.

We report four cases of nonvaccinated SARS-CoV-2-positive pregnant women that had a stillbirth in our tertiary centre below.

### 1.3. Patient 1

The patient is a 27-year-old healthy multipara, BMI 23.4 kg/m^2^ with two previous term pregnancies complicated by late fetal growth restriction. The pregnancy follow-up was performed at a primary care unit with no complications reported during gestation. The third-trimester ultrasound revealed a normally growing fetus (percentile 50) with normal amniotic fluid levels. At 39 weeks of gestation, the patient entered the emergency department with absent fetal movements and irregular uterine contractions. On admission, a scan confirmed the diagnosis of in utero fetal demise. Her blood pressure was 170-110 mmHg, and her blood and urine test results revealed 21 × 10^9^/L platelets, uric acid 8.7 mg/dL, AST 50 unit/L, ALT 31 unit/L, LDH 478 unit/L, CRP 2.42 mg/dL, and 400 mg/dL proteinuria. She was diagnosed with severe preeclampsia and was admitted for labour induction. A PCR-SARS-CoV-2 test was performed upon admission and resulted positive. She was asymptomatic for COVID-19. Severe thrombocytopenia was managed with Dexamethasone. Labetalol was used to lower her blood pressure levels. Labour was induced with prostaglandin E1. She had a vaginal delivery of a morphologically normal fetus, and no visible changes were seen in the amniotic fluid, the membranes, or in the umbilical cord. The fetus tested negative on a PCR-SARS-CoV-2 test performed at delivery. A fetal autopsy revealed a normal fetus with a low birth weight—2050 grams (percentile 0.1). Placental histologic examination showed several signs of maternal vascular malperfusion, infarctions on 60% of the placental surface and chorangioma.

### 1.4. Patient 2

25-year-old healthy nullipara, BMI 22.4 kg/m^2^, had her pregnancy follow-up at a private hospital. No complications were reported during gestation until week 34, when a routine scan revealed a light weight for gestational age fetus—percentile 7, with normal amniotic fluid volume and normal doppler examination. At week 35 and 4 days, the patient presented to the emergency department due to absent fetal movements. In utero fetal demise was confirmed with a scan, and she was admitted for labour induction. Her blood test results revealed 14.7 × 10^9^/L leukocytes and 1.98 mg/dL CRP. She tested positive for SARS-CoV-2 on a PCR screening test performed upon admission, and she was asymptomatic for COVID-19. Labour was induced with prostaglandin E1, and she had a vaginal delivery of a morphologically normal fetus with low birth weight—1960 grams (percentile 2). The fetus tested positive on a PCR-SARS-CoV-2 test. There were no visible changes in the amniotic fluid, the membranes, or in the umbilical cord. Placental histologic examination revealed increased intravillous lymphocytes and fibrin, avascular villi, and syncytial knots, signs that are suggestive of maternal vascular malperfusion.

### 1.5. Patient 3

The patient is a 31-year-old healthy multipara, BMI 34 kg/m^2^ with two previous pregnancies, the latest complicated by severe preeclampsia with thrombocytopenia at 40 weeks of gestation. She had her pregnancy follow-up at a primary care unit, and her 3^rd^-trimester ultrasound revealed a normally growing fetus (percentile 50) with normal amniotic fluid level. At 36 weeks of gestation, her general practitioner referred her to our outpatient clinic due to elevated blood pressure levels in consecutive measurements (≥140-90 mmHg). At week 38 and 1 day, she had her first appointment in our hospital; her blood pressure levels were normal, she did not have proteinuria, and antepartum fetal monitoring was reassuring. Four days later, she presented to the emergency department, at week 38 and 5 days, with absent fetal movements and uterine contractions. A scan performed upon admission confirmed in utero fetal demise. She was admitted for labour induction and was tested for SARS-CoV-2 as part of the preadmission screening protocol. She tested positive and was asymptomatic for COVID-19. Her blood and urine test results revealed 109 × 10^9^/L platelets, 1.98 mg/dL CRP, and 500 mg/dL leukocyturia. Labour was induced with prostaglandin E1, and she had a vaginal delivery of a morphologically normal fetus with a negative fetal PCR-SARS-CoV-2 result. There were no visible changes in the amniotic fluid, the membranes, or the umbilical cord. Fetal autopsy revealed a fetus weighing 2300 grams (percentile 1) with no malformations. Placental histologic examination revealed mild acute chorioamnionitis and signs of fetal vascular malperfusion.

### 1.6. Patient 4

33-year-old healthy multipara (one previous normal pregnancy), BMI 25.7 kg/m^2^, with a spontaneous uncomplicated dichorionic twin gestation had her pregnancy follow-up in a private hospital. At 30 weeks and 3 days of gestation, she presented to the emergency department with suspected preterm premature rupture of membranes (PPROM). On admission, she reported fever and cough within one day of evolution. She was normotensive and febrile—38.1°C. Physical examination confirmed PPROM of the first fetus with clear amniotic fluid. Obstetric ultrasound showed the first fetus in breech presentation and the second fetus in transverse presentation, both with cardiac activity. She was admitted for PPROM management, and both fetuses were monitored continually with cardiotocography. She tested positive in a SARS-CoV-2 PCR test. Laboratory test results showed Hb 14.2 g/dL, platelets 62 × 10^9^/L, APTT 57.6 seconds (control 28 seconds), prothrombin time 16.0 seconds (control 11.5 seconds), fibrinogen 56 mg/dl, AST 280 unit/L, ALT 114 unit/L, total bilirubin 1.0 mg/dl, LDH 1460 unit/L, and spot urine protein/creatinine ratio of 2.6. These values were compatible with HELLP syndrome and disseminated intravascular coagulation (DIC). She was promptly transferred to a COVID-19 isolation unit, and both fetuses were demonitorised for a short period. At the COVID-19 unit, due to difficulties in remonitoring the second fetus, a scan was performed, revealing in utero fetal demise. Maternal status deterioration and nonreassuring fetal heart rate of the first fetus justified an emergent caesarean section that was performed six hours after admission. She delivered a live newborn with an Apgar score of 2-4-5, weighing 1270 grams (percentile 4), followed by a stillborn. There were no macroscopic changes detected in both newborn and fetus, nor in both membranes and umbilical cords. The live-born baby presented a blood panel that was similar to his mother's and ended up dying on his second day of life. Both babies tested positive for SARS-CoV-2. The mother was transferred to an intensive care unit for DIC and HELLP syndrome management. She was discharged on day 10 postpartum with a good recovery. Placental histologic examination revealed infarctions, increased intervillous fibrin, avascular villi, intervillous haemorrhagic thrombi, and histiocytic intervilitis. Chorangioma was detected in the placenta of the second fetus.

All women presented in this case series expressed their wish to contribute to the pool of data on maternal and perinatal outcomes of COVID-19 in pregnancy. Their aim was to improve guidance and management of other pregnant women during the current pandemic.

## 2. Discussion

To our knowledge, this is the first case series of stillbirths in women infected by SARS-CoV-2 in pregnancy in a Portuguese cohort. The main findings of our series are as follows: the increased rate of stillbirth in our centre from April 2020 to March 2021 0.52% (14/2680) compared to the analogous period of the 4 previous years; the increased rate of stillbirth cases among SARS-CoV-2-infected pregnant women 3.84% (5/130); the increased incidence of obstetric conditions related to placental vascular malperfusion among stillbirths that occurred in SARS-CoV-2-infected pregnancies 60.00% (3/5); the increased incidence of low birth weight among stillbirths of mothers with COVID-19 60.00% (3/5); the majority 60.00% (3/5) of infected women who had a stillbirth were asymptomatic for COVID-19; and the high incidence of a positive PCR-SARS-CoV-2 test 60.00% (3/5) among stillbirths of mothers with COVID-19.

In recent pregnant women studies, stillbirth has more commonly been reported as a perinatal outcome of SARS-CoV-2 infection [9, 16, 22]. Due to pandemic restrictions and health services overload, several cohorts have seen an increase in stillbirth rates among positive and negative mothers [26, 27]. Our findings are consistent with what has been reported in literature—we have had a higher stillbirth rate in our centre from April 2020 until March 2021 compared to the same period in the four previous years. In the period of April 2016-March 2017, we notice the second-highest stillbirth rate in the last 5 years. This was due to an elevated number of cases of fetal hydrops (3/11) and infection (3/11) during that period. The authors assume these events as outliers since the number of fetal hydrops and infection in our centre in the last 5 years was approximately 0 and 1 cases per year, respectively.

**Figure 1:**
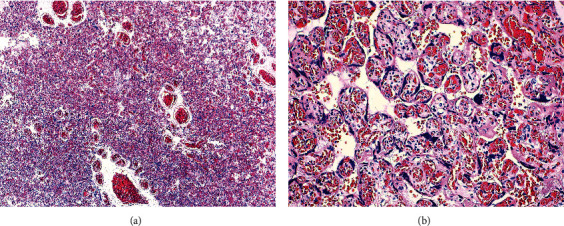
(a, b) Gross dissection revealed a firm area which measured 3 cm that histologically corresponded to a proliferation of capillary sized vessels causing expansion of contiguous affected villi, composed of endothelial cells, pericytes, and myofibroblastic stromal cells, compatible with the diagnosis of chorangioma (hematoxylin-eosin stain, original magnification 4x on the left and 20x on the right).

**Figure 2:**
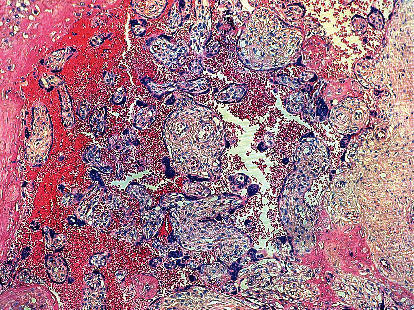
Haemorrhage and intravillositary thrombus, compatible with maternal vascular malperfusion (hematoxylin-eosin stain, original magnification 10x).

**Figure 3:**
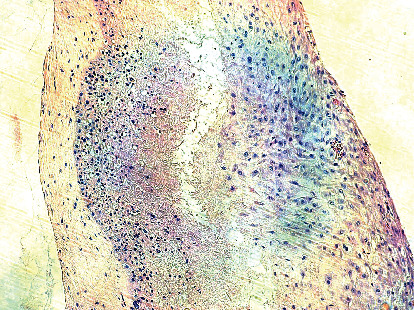
Membrane inflammatory infiltrate rich in lymphocytes (hematoxylin-eosin stain, original magnification 10x).

Regarding stillbirth cases that occurred between April 2020 and March 2021, the incidence of this outcome was much greater in the SARS-CoV-2-positive group 3.84% (5/130) compared to the SARS-CoV-2 negative group 0.35% (9/2550) (*p* < 0.05). This observation has been widely seen in other cohorts [9, 16, 22], and researchers have been pointing at the placenta as a possible explanation for the increased risk of fetal death in infected pregnant patients. The potential mechanism might be primarily explained on the basis of a secondary effect of the virus owing to placental hypoperfusion [24]. SARS-CoV-2 infection is accompanied by an aggressive inflammatory response with the release of a large number of proinflammatory cytokines in an event known as “cytokine storm” [21]. This response causes endothelial damage and triggers coagulation abnormalities [28–30], which compromise the perfusion of multiple organs including the placenta. Several reports have described placental vascular malperfusion findings in patients with COVID-19 [23, 24]. Most commonly described features are signs of maternal vascular malperfusion such as intervillous thrombi, signs of fetal vascular malperfusion, and chorangiosis [23, 24, 31]. Placental histopathologic exams of the cases from our series are concordant with what has been reported in the literature. All stillbirth placentas presented signs of maternal and fetal malperfusion, such as infarctions, intervillous thrombi, and chorangioma, as seen in Figures 1–3.

In our series, 3 out of 5 women in the SARS-CoV-2-positive group developed obstetric conditions related to placental hypoperfusion such as preeclampsia and HELLP syndrome. The events occurred in a higher, but not statistically different proportion compared to the SARS-CoV-2 negative group (3 (60%) vs. 1 (11.1%); *p* < 0.09). Although it is known that these conditions themselves increase the risk of adverse obstetric outcomes such as stillbirth, it is also known that they have been increasingly reported as outcomes of SARS-CoV-2 infection in pregnancy [10, 32–34]. Until the date of this case series report, the association between preeclampsia/HELLP syndrome and COVID-19 is still under study as there are multiple common clinical features of preeclampsia and systemic SARS-CoV-2 infection that can hamper their differential diagnosis [35]. Out of 5 stillbirths from SARS-CoV-2-positive mothers, 3 had low birth weight compared to those of the same gestational age. This suggests that intrauterine fetal growth restriction might have taken place during gestation. Regardless of these pregnancies being at higher risk of poor maternal outcomes, we hypothesize that SARS-CoV-2 infection might have been a trigger or an adjuvant factor to the cascade of events that culminated in fetal death.

Interestingly, in 3 out of 4 cases we report in this series, patients were asymptomatic for COVID-19. Given the literature suggests that increasing COVID-19 severity is related to worse maternal outcomes [22, 25], our sample did not seem to follow this pattern. This should raise awareness among clinicians and patients as the majority of pregnant women are asymptomatic or develop mild COVID-19 symptoms [3].

Finally, 3 out of the 5 fetuses tested positive for SARS-CoV-2 on a PCR test performed at birth. This finding is also surprising given that vertical transmission seems to occur in a minority of cases [36] since the placenta lacks the expression of proteins required for SARS-CoV-2 infection [37].

Our case report adds further information to the scientific community, patients, and clinicians about perinatal consequences of SARS-CoV-2 infection in pregnancy. An important observation of our study—that asymptomatic pregnant women had the worst perinatal consequence, a stillbirth—is of uttermost importance to be reported as it may guide clinical decisions, prenatal counselling, and development of public health measures such as vaccination policies and protection of vulnerable groups.

The strengths of our study are complete placental histologic examinations, performed by the same team of pathologists, allowing for better interpretation of the mechanisms behind fetal death. The major limitation of this case series is the small sample size which hinders our ability to draw conclusions beyond this initial observation.

## 3. Conclusion

Our case series reports stillbirth as an outcome of maternal SARS-CoV-2 infection. The viral immune response seems to play a role in the cascade of events that lead to placental hypoperfusion and consequently compromise fetal well-being. Stillbirth can occur in COVID-19 asymptomatic pregnant women. This should raise awareness among clinicians and patients. We urge more studies to explore the association between SARS-CoV-2 infection and the risk of stillbirth.

## Figures and Tables

**Table 1:** Maternal and pregnancy characteristics of stillbirth cases that occurred in a tertiary centre in Portugal between April 2020 and March 2021.

		All stillbirths (*N* = 14)	SARS-CoV-2 negative (*N* = 9)	SARS-CoV-2 positive (*N* = 5)	*p* value (*χ*^2^ test)
Maternal age		30.21 (5.78)	31.22 (6.72)	28.40 (3.43)	0.31
Parity	Nulliparous	5 (35.71%)	4 (44.44%)	1 (20.00%)	0.58
	Multiparous	9 (64.28%)	5 (55.55%)	4 (80.00%)	
Origin	African	3 (21.42%)	1 (11.11%)	2 (40.00%)	0.68
	Asian	1 (7.14%)	1 (11.11%)	0	
	European	10 (71.42%)	7 (77.77%)	3 (60.00%)	
Pregnancy follow up	Primary care	5 (35.71%)	3 (33.33%)	2 (40.00%)	0.80
	Tertiary hospital	5 (35.71%)	4 (44.44%)	1 (20.00%)	
	Private hospital	4 (28.57%)	2 (22.22%)	2 (40.00%)	
Relevant medical history		2 (14.28%)	2 (22.22%)	0	0.50
Of which, chronic hypertension		1 (7.14%)	1 (11.11%)	0	1
Relevant obstetric history		6 (42.85%)	3 (33.33%)	3 (60.00%)	1
Of which, preeclampsia		2 (14.28%)	1 (11.11%)	1 (20.00%)	0.50
Of which, fetal growth restriction		1 (7.14%)	0	1 (20.00%)	
Obstetric outcomes in the current pregnancy	Preeclampsia/HELLP syndrome	4 (28.57%)	1(11.11%)	3 (60.00%)	0.09
	Gestational diabetes	2 (14.28%)	2 (22.22%)	0	0.50
	Hydramnios	2 (14.28%)	2 (22.22%)	0	0.50
	Rupture of membranes	1 (7.14%)	1 (11.11%)	1 (20.00%)	1
	Fetal-maternal haemorrhage	1 (7.14%)	1 (11.11%)	0	1
Stillbirth gestational age		34.29 (4.61)	33.67 (5.22)	35.40 (3.50)	0.47
Stillbirth weight (centile)	Normal birth weight	10 (71.42%)	9 (100.00%)	1 (20.00%)	0.01
	Low birth weight	3 (21.42%)	0	3 (60.00%)	
	Unknown	1 (7.14%)	0	1 (20.00%)	
Mode of delivery	Vaginal	9 (64.28%)	6 (66.66%)	3 (60.00%)	1
	Caesarean	5 (35.71%)	3 (33.33%)	2 (40.00%)	

**Table 2:** Five-year analysis of stillbirth rate in a tertiary centre in Portugal.

Period	Total fetal deaths	Total newborns	Stillbirth rate	*Z*-test (*p* value)
April 2020–March 2021	14	2680	0.52%	
April 2019–March 2020	8	2883	0.27%	0.14
April 2018–March 2019	8	2889	0.27%	0.14
April 2017–March 2018	8	2822	0.28%	0.16
April 2016–March 2017	11	2754	0.39%	0.50
